# Incidence and Characteristics of Cataract Surgery in Poland, during 2010–2015

**DOI:** 10.3390/ijerph15030435

**Published:** 2018-03-02

**Authors:** Michał S. Nowak, Iwona Grabska-Liberek, Katarzyna Michalska-Małecka, Andrzej Grzybowski, Milena Kozioł, Wojciech Niemczyk, Barbara Więckowska, Jacek P. Szaflik

**Affiliations:** 1 Provisus Eye Clinic, 112 Redzinska str., 42-209 Czestochowa, Poland; 2 Saint Family Hospital Medical Center, 19 Wigury str., 90-302 Lodz, Poland; 3 Department of Ophthalmology, Centre of Postgraduate Medical Education, 231 Czerniakowska str., 01-416 Warsaw, Poland; iliberek@gmail.com; 4 Department of Ophthalmology, School of Medicine in Katowice, Medical University of Silesia, 35 Ceglana str., 40-952 Katowice, Poland; k.michalska.malecka@gmail.com; 5 Department of Ophthalmology, University of Warmia and Mazury, 30 Warszawska str., 10-082 Olsztyn, Poland; ae.grzybowski@gmail.com; 6 Institute for Research in Ophthalmology, Foundation for Ophthalmology Development, Gorczyczewskiego 2/3, 60-554 Poznan, Poland; 7 Department of Analyses and Strategy, Polish Ministry of Health, 15 Miodowa str., 00-952 Warsaw, Poland; m.koziol@mz.gov.pl (M.K.); w.niemczyk@mz.gov.pl (W.N.); 8 Department of Social Insurance, Warsaw School of Economics, 162 Niepodleglosci Ave., 00-001 Warsaw, Poland; bawie@sgh.waw.pl; 9 Department of Ophthalmology, SPKSO Ophthalmic Hospital, Medical University of Warsaw, 13 Sierakowskiego str., 03-709 Warsaw, Poland; jacek@szaflik.pl

**Keywords:** eye, cataract, epidemiology, public health

## Abstract

**Background:** To assess the incidence and characteristic of cataract surgery in Poland from 2010 to 2015 and to interpret these findings. **Patients and methods:** Data from all patients who underwent cataract surgery alone or in combined procedures in Poland between January 2010 and December 2015 were evaluated. Patient data were from the national database of hospitalizations maintained by National Health Fund. Data on the population of Poland were obtained from Central Statistical Office of Poland. **Results:** In total, 1,218,777 cataract extractions (alone or combined with other procedures) were performed in 1,081,345 patients during 2010–2015. Overall, the incidence of cataract surgery increased from 5.22/1000 person-years in 2010 to 6.17/1000 person-years in 2015. Phacoemulsification was performed in 97.46% of cataract extractions, and 3.02% of cataract extractions were combined procedures. The rate of one-day procedures increased from 28.3% in 2010 to 43.1% in 2015. The probability of second-eye surgery 12 months after the first-eye surgery increased from 44% in 2010 to 73% in 2015 (log-rank test *p* < 0.0001). **Conclusion:** In Poland, from 2010 to 2015, the total incidence of cataract surgery, the number of people who underwent surgery, and the number of one-day cataract surgeries increased significantly.

## 1. Introduction

According to the Vision Loss Expert Group, cataract was the leading cause of blindness and the second major cause of moderate and severe visual impairment (MSVI) in 2010. Although the percentage of global blindness and MSVI caused by cataract decreased from 38.6% to 33.4% and from 25.6% to 18.4%, respectively, in 20 years (from 1990 to 2010), barriers to uptake of cataract surgery still exist in most countries [1,2]. These reports, had a lack of data from the Eastern European region, including Poland. Poland is the largest eastern European country, with a population of 38 million people in 2011 [3]. Recently published reports by Nowak and Smigielski [4,5] showed that retinal diseases are the major cause of blindness and visual impairment in Poland, as in Western European countries. These reports also revealed that cataract is the second major cause of vision loss in Poland, affecting more than 20% of people aged 60 years or older.

Globally, the total number of cataract surgeries has increased in all regions, especially in Asia [2]. However, reports on the incidence of cataract surgery based on national basis are limited. In 2011, Behndig et al. [6] reported one million cataract surgeries in Swedish National Cataract Register from 1992 to 2009. In 2015, Daien et al. [7] reported the incidence of cataract surgery in France from 2009 to 2012 was 2.7 million cases. Szigiato et al. [8] published data in 2016 from the Ontario Health Insurance Plan regarding cataract surgeries performed in 2000–2013 in Ontario, Canada. In Poland, health services are free of charge for every citizen and guaranteed by the government and the Polish constitution. The National Health Fund (Narodowy Fundusz Zdrowia (NFZ)) maintains the national database of hospitalizations, which records all medical procedures and provides accurate population-based medical data.

To analyze these data, the Polish Ministry of Health implemented a project titled “Maps of Healthcare Needs—Database of Systemic and Implementation Analyses,” which was co-financed by the European Union funds through the European Social Fund under the Operational Program of Knowledge, Education and Development. Our study was part of this project. The aim of the present study was to assess the incidence and characteristic of cataract surgery in Poland from 2010 to 2015 and to interpret these findings.

## 2. Material and Methods

The study design was a retrospective population-based survey. The data from all patients who underwent cataract surgery alone or in combined procedures in Poland between January 2010 and December 2015 were assessed from the national database of hospitalizations [9]. This database is maintained by NFZ, which compiles all data related to hospitalizations in public and private hospitals financed from public sources. The information includes medical data, identification number, date of birth, area code, and sex of patients. The medical data include the diagnoses coded according to the International Classification of Diseases, 10th Revision, and all procedures performed coded using the International Classification of Diseases, 9th Revision (ICD-9) procedure codes and unique NFZ codes corresponding to certain hospital procedures.

For each individual patient, cataract surgery alone or as a combined procedure with corneal transplantation, glaucoma filtrating surgery, or vitrectomy was retrospectively identified. The ICD-9 code 13.4 was used to identify cataract extraction performed by phacoemulsification, with 13.2, 13.3 and 13.5 codes used to identify other types of cataract extractions. The following NFZ codes were used: B12, B13, B14, B15, B18, and B19 corresponding to cataract surgery alone; B04, B05, and B06 corresponding to cataract surgery combined with corneal transplantations; B11 corresponding to cataract surgery followed by glaucoma filtrating surgery; and B16 and B17 corresponding to cataract surgery combined with vitrectomy. The number of one-day procedures was also obtained from the NFZ data. The wait time data and the number of patients waiting for cataract surgery were obtained from the national registry [10].

For statistical analysis, the socio-demographic data of cataract patients including age, sex and place of residence were anonymously recorded. Data regarding the population of Poland were obtained from Central Statistical Office of Poland (Głowny Urzad Statystyczny) [11]. The incidence of cataract surgery was presented for each year separately and by age category matched with corresponding year population data in Poland. The statistical analysis also included the annual volume of cataract surgery, calculations of cataract extractions performed by phacoemulsification, calculations of proportions of one-day and combined procedures, and data regarding the number of cataract surgeries received by Polish patients in other European Union countries. The number of patients having cataract surgery in the second eye was collected, and the wait times were calculated. The Kaplan–Meier method was used to calculate the cumulative probability of second-eye cataract surgery during 2010–2015 with a log-rank test to compare the curves between periods. The demographic characteristics of patients are presented with the mean and standard deviation (SD). The study protocol was approved by the Polish Ministry of Health.

## 3. Results

**Figure 1:**
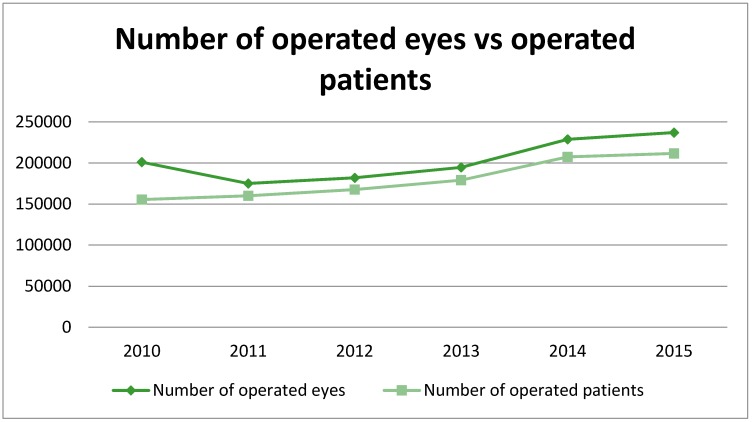
The number of operated eyes vs. operated patients in Poland in 2010–2015.

Overall, the incidence of cataract surgery in Poland increased from 5.22/1000 person-years in 2010 to 6.17/1000 person-years in 2015 (Table 1). The incidence of cataract surgery ranged from 0.03/1000 person-years in children to 40.92/1000 person-years in people aged 70 years and older. In total, 1,218,777 cataract extractions (alone or combined with other procedures) in 1,081,345 patients were performed during 2010–2015 (Table 2 and Figure 1).

The number of operated eyes increased by 17.9% from 201,083 in 2010 to 237,098 in 2015. However, during 2011–2013, a significant decrease in the number of cataract extractions was observed. In contrast to this finding, the number of operated patients steadily increased between 2010 and 2015 from 155,409 to 211,637, respectively (Figure 1). In the study period, women represented 65.1% of patients (Table 1 and Table 3), and the mean age at surgery was 73.2 ± 10.2 years. Young males had relatively more cataract percentage, probably due to traumatic cataract, while older females (i.e., >70 years old) had higher cataract surgery population due to the total higher female population as a result of women’s longer life expectancy.

Distribution of cataract patients according to the place of residence is presented in Table 3. In Poland 74.6% of patients who underwent cataract surgery during 2010–2015 lived or had lived in urban areas. However, the availability of cataract surgery for rural residents slightly increased in this period from 24.2% in 2010 to 27.9% in 2015. Characteristics of cataract surgery in Poland from 2010 to 2015 are presented in Table 2.

**Figure 2:**
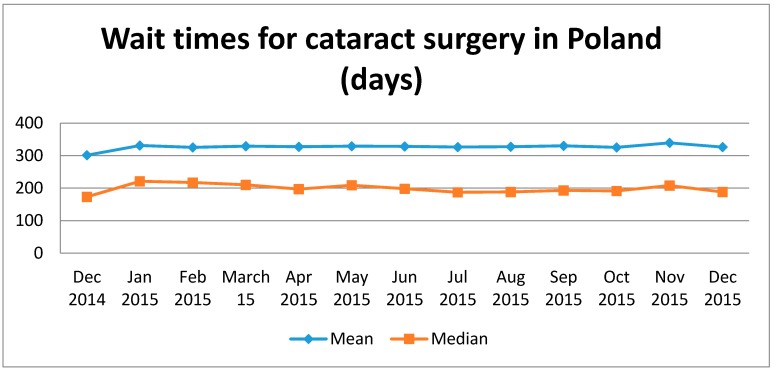
Wait times for cataract surgery in Poland.

**Figure 3:**
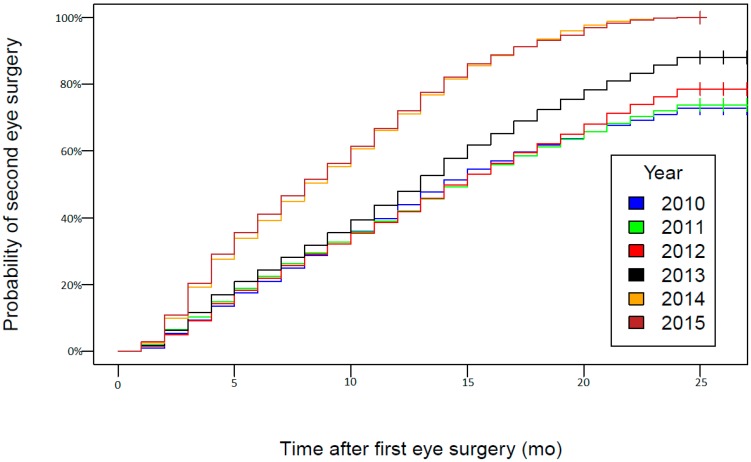
The Kaplan–Meier analysis showing the probability of second-eye surgery in Poland in 2010–2015.

Phacoemulsification was performed in 97.46% of cataract extractions. The use of extracapsular lens extraction decreased from 3.84% in 2010 to 1.65% in 2015. During the analyzed period, 3.02% of cataract extractions were combined procedures. The number of combined procedures increased by 15% between 2010 and 2015, and the highest number was observed in 2014. The majority of cataract extractions were combined with vitrectomy (61.77%), followed by glaucoma filtrating surgery (35.55%) and corneal transplantation (2.68%). In total, 22,755 phacovitrectomies were performed during 2010–2015, which represented 1.87% of all cataract procedures in this period. Cataract extractions combined with glaucoma surgery were reported in 13,094 eyes (1.07% of all cataract procedures during 2010–2015), and corneal transplants combined with cataract surgery were performed in 986 eyes (0.08% of all cataract procedures during 2010–2015). In the study period, 37.1% of cataract surgeries were one-day procedures in Poland (Table 2). However, the rate of one-day procedures significantly increased from 28.3% in 2010 to 43.1% in 2015. Nationwide data on the number of people waiting for cataract surgery and on wait times in Poland were available from December 2014. In total, 539,019 persons were waiting for cataract surgery in December 2015, and this number had increased by 7% from December 2014. These numbers mean that mean and median wait times for cataract surgery on December 2015 were 326 and 188 days, respectively (Figure 2). However, the probability of second-eye surgery 12 months after the first-eye surgery increased from 44% in 2010 to 73% in 2015 (log-rank test *p* < 0.0001). The Kaplan–Meier analysis showing the probability of second-eye surgery in Poland between 2010 and 2015 is presented in Figure 3.

In November 2014, the Polish government introduced the European Union Directive on the application of patients’ rights on cross-border health care. From November 2014 to December 2015, 4542 Polish patients had cataract surgery in other European Union countries [12]. Ninety-five percent of them had cataract surgery in Czech Republic, and the remaining patients received care in Germany, Latvia, and Slovak Republic.

## 4. Discussion

This study provides for the first time data concerning the incidence and characteristics of cataract surgery from the Eastern European region. The study describes trends of cataract surgery and age-specific rates of the incidence of cataract surgery in the overall population of Poland during 2010–2015. During the study period, the incidence of cataract surgery in Poland increased from 5.22/1000 person-years in 2010 to 6.17/1000 person-years in 2015, with a significant decrease during 2011–2013. This decrease was mainly attributable to the reduction in the reimbursement cost of cataract surgery procedure from the NFZ for public and private hospitals. The hospitals had to reorganize their ophthalmology departments and cut the surgery costs. Afterwards, a significant increase in the number of cataract extractions was observed. Our trends agree with the results of previous studies from Western countries that showed a significant increase in the incidence of cataract surgery over time. The incidence of cataract surgery in Sweden increased from 4.47/1000 person-years in 1992 to 9.0/1000 person-years in 2009 [6]. In comparison, the incidence of cataract surgery in Ontario increased from 7.3/1000 person-years in 2000 to 10.5/1000 person years in 2012; in Minnesota, US, from 8.5/1000 person-years in 2005 to 11.0/1000 person-years in 2011; and, in France, from 9.86/1000 person-years in 2009 to 11.08/1000 person-years in 2012 [7,8,13]. The overall incidence of cataract surgery per 1000/person-years in Poland was significantly lower than in Sweden, France, Canada, and the United States. However, strong associations of socioeconomic indices with quantity and quality of cataract surgery persists throughout the world [2,14]. Poland is a middle-income country and spends much less money on health care than Western countries [11]. Nevertheless, we found that the probability of second-eye surgery 12 months after the first-eye surgery increased during the research period from 44% to 73%, which is higher than in France but lower than in the United States [7,13]. In the present analysis, women represented 65.1% of the patient population. Other studies also noted a difference in surgical rate by sex. The proportion of women who underwent cataract extraction was 61% in Sweden, 59% in France, and it was also significantly higher than in men in Minnesota [6,7,13]. Globally, women have higher rates of blindness and moderate and severe visual impairment (MSVI) caused by cataract compared with men, and sex inequity in cataract surgery persists in low- and middle-income countries, where men are 1.71 times more likely to have cataract surgery than women [2]. Despite the decline in the prevalence of blindness and MSVI caused by cataract, it was still the main cause of blindness and the second most common cause of MSVI in 2010 [1,2]. This outcome was mainly the result of the rapid aging of populations [2,7]. In the present study and in previously published studies, the incidence of cataract surgery increased in older age groups [7,15,16,17]. In Poland, only 0.1% of all cataract surgeries were pediatric cases. In the present study the mean age at cataract surgery was 73.2 ± 10.2 years. In other studies, the mean age for cataract surgery was 73.4 years in France in 2012, 73.0 years in Minnesota in 2011, and 74.9 years in Sweden in 2009 [6,7,13]. During the study period, a low number of cataract surgeries were performed as a one-day procedure in Poland. During 2010–2015, only 37.1% of cataract surgeries were one-day procedures, which placed Poland, together with Romania, Croatia and FYR of Macedonia, among the OECD countries with the lowest number of one-day procedures in cataract surgery [18]. At the same time, in many Western European countries and in some Eastern European countries, such as Estonia and Slovenia, the rate of one-day procedures in cataract surgery was over 95% [18]. However, the rate of one-day procedures in Poland significantly increased from 28.3% in 2010 to 43.1% in 2015, which is a desired trend. No bilateral cataract surgery was officially reported during 2010–2015 in the NFZ database, mainly due to lack of legal regulations in this matter, which were introduced in January 2017. The use of phacoemulsification occurred in 97.46% of cataract extractions in Poland in 2010–2015. The use of this technique was lower than in France, but it was significantly higher than in other low- and middle-income countries [7,19]. The use of extracapsular lens extraction decreased from 3.84% in 2010 to 1.65% in 2015; however, it remains useful for some patients. The total number of cataract extractions combined with other procedures (vitrectomy, glaucoma filtrating surgery and corneal transplantation) in Poland increased by 15% between 2010 and 2015. It is likely that these increases might have been related to the changes in the approach to vitreoretinal diseases. Combined phacovitrectomy has been reported to be significantly less costly to Medicare in the United States than a two-step approach for patients with an indication for vitrectomy and a visually significant cataract [20]. During the study period, 74.6% of patients who underwent cataract surgery in Poland lived in urban areas. A barrier for cataract surgery for rural residents was the low distribution of ophthalmologists across some regions. Other barriers for regularly performing cataract surgery in Poland included insufficient financing from the NFZ and other social, infrastructural, and geographic factors, which resulted in patients seeking care elsewhere in the country as well outside Poland [12]. From November 2014 to December 2015, 4542 Polish patients had cataract surgery in other European Union countries. All of them had their surgery costs reimbursed by Polish NFZ due to the European Union Directive on the application of patients’ rights on cross-border healthcare [12].

Limitations of the present study include possible presence of misclassification or biases related to under-detection. Errors in using specific ICD-9 and NFZ codes might have occurred at different levels (operating theaters, hospitals, and NFZ offices), but such mistakes likely had only a minor impact on the study findings. Our study was country based and covered the overall population of Poland. The population size, national recruitment and impact of its findings on public health are the most important strengths of the current study. However, our results are specific to the Polish health care system only and cannot describe other health care systems in Eastern Europe. It would be also beneficial to have data on the outcomes of cataract surgery in the future, because nowadays these data are not collected in the national database of hospitalizations.

## 5. Conclusions

In conclusion, this study reported the incidence and characteristics of cataract surgery in the overall population of Poland during 2010–2015. There were very positive trends in cataract surgery during these years. Despite the decrease in the number of operated eyes during 2011–2013, from 2010 to 2015 the total incidence of cataract surgery significantly increased, as did the number of people who underwent surgery, and the number of one-day cataract surgeries. In addition, the probability of second-eye surgery 12 months after the first-eye surgery increased from 44% in 2010 to 73% in 2015. Nevertheless, few Polish patients had cataract surgery abroad.

## Figures and Tables

**Table 1:** Incidence of cataract surgery in Poland from 2010 to 2015 by age group.

	2010	2011	2012	2013	2014	2015
No. age 0–18 years	7643553	7630880	7531582	7431731	7367066	7309001
No. of cataract Surgery	245	267	272	294	270	242
Incidence/1000 person-years Gender, % Women	0.03	0.03	0.04	0.04	0.04	0.03
	35.9%	41.6%	45.6%	44.2%	46.7%	45.5%
No. age 19–39 years	12482309	12523386	12461398	12355235	12201430	12015345
No. of cataract Surgery	1441	1305	1280	1324	1383	1384
Incidence/1000 person-years Gender, % Women	0.12	0.10	0.10	0.11	0.11	0.12
	43.9%	40.8%	40.5%	41.5%	41.9%	41.5%
No. age 40–49 years	4792211	4822159	4838436	4879816	4956005	5064587
No. of cataract Surgery	3007	2680	2620	2583	2849	2920
Incidence/1000 person-years Gender, % Women	0.63	0.56	0.54	0.53	0.57	0.58
	47.4%	47.1%	47.4%	47.9%	48.4%	49.4%
No. age 50–59 years	5770823	5765460	5656651	5536118	5406320	5245352
No. of cataract Surgery	15598	13397	13203	13188	14645	14001
Incidence/1000 person-years Gender, % Women	2.70	2.32	2.33	2.38	2.71	2.67
	50.6%	51.6%	51.6%	51.5%	50.7%	51.4%
No. age 60–69 years	3682048	3931289	4171206	4409809	4642821	4888294
No. of cataract Surgery	38973	35322	37986	43120	52747	57646
Incidence/1000 person-years Gender, % Women	10.59	8.98	9.11	9.78	11.36	11.79
	60.2%	59.7%	59.8%	59.7%	59.6%	59.9%
No. age ≥70 years	4146056	3852826	3874727	3889291	3910358	3932421
No. of cataract Surgery	141819	122035	126644	134212	156970	160905
Incidence/1000 person-years Gender, % Women	34.21	31.67	32.68	34.51	40.14	40.92
	69.1%	68.9%	68.5%	68.6%	68.8%	68.4%
No. all	38517000	38526000	38534000	38502000	38484000	38455000
No of cataract Surgery	201083	175006	182005	194721	228864	237098
Incidence/1000 person-years Gender, % Women	5.22	4.54	4.72	5,06	5.95	6.17
	65.4%	65.1%	64.9%	65.0%	65.1%	64.9%

**Table 2:** Characteristics of cataract surgery in Poland from 2010 to 2015.

	2010	2011	2012	2013	2014	2015	All
No. of cataract surgery by surgical technique phacoemulsification (%)	193,362 (96.16%)	169,632 (96.93%)	177,152 (97.33%)	190,005 (97.58%)	224,491 (98.09%)	233,185 (98.35%)	1,187,827 (97.46%)
No. of cataract surgery by surgical technique extracapsular extraction (%)	7721 (3.84%)	5374 (3.07%)	4853 (2.67%)	4716 (2.42%)	4373 (1.91%)	3913 (1.65%)	30,950 (2.54%)
No. cataract surgery combined with corneal transplantation (%)	114 (0.06%)	186 (0.11%)	182 (0.10%)	159 (0.08%)	187 (0.08%)	158 (0.07%)	986 (0.08%)
No. cataract surgery combined with glaucoma filtration surgery (%)	2278 (1.13%)	2002 (1.14%)	2175 (1.19%)	2286 (1.17%)	2304 (1.01%)	2049 (0.86%)	13,094 (1.07%)
No. cataract surgery combined with pars plana vitrectomy (%)	2884 (1.43%)	3199 (1.83)	3732 (2.05%)	4211 (2.16%)	4381 (1.91%)	4348 (1.83%)	227,55 (1.87%)
No. of one-day procedures	56,870 (28.28%)	59,700 (34.11%)	67,183 (36.91%)	72,218 (37.09%)	93,495 (40.85%)	102,222 (43.11%)	451,688 (37.06%)

**Table 3:** Demographic characteristics of patients who underwent cataract surgery in Poland from 2010 to 2015.

	2010	2011	2012	2013	2014	2015	All
Age mean ± SE	73.1 ± 10.3	73.0 ± 10.4	73.2 ± 10.4	73.2 ± 10.3	73.4 ± 10.1	73.4 ± 10.0	73.2 ± 10.2
Women	131,548	113,938	118,108	126,564	148,949	153,913	794,020
(%)	(65.42%)	(65.10%)	(64.89%)	(65.00%)	(65.08%)	(64.91%)	(65.15%)
Men	69,535	61,068	63,897	68,157	79,915	83,185	424,757
(%)	(34.58%)	(34.90%)	(35.11%)	(35.00%)	(34.92%)	(35.09%)	(34.85%)
Urban residence	152,418	130,039	134,501	143,495	166,508	171,005	897,966
(%)	(75.80%)	(74.30%)	(73.90%)	(73.70%)	(72.75%)	(72.12%)	(74.58%)
Rural residence	48,665	44,967	47,504	51,226	62,356	55,093	309,811
(%)	(24.20%)	(25.70%)	(26.10%)	(26.30%)	(27.25%)	(27.88%)	(25.42%)
